# Polysaccharides extracted from mulberry fruits (*Morus nigra* L.): antioxidant effect of ameliorating H_2_O_2_-induced liver injury in HepG2 cells

**DOI:** 10.1186/s12906-023-03925-w

**Published:** 2023-04-12

**Authors:** Xinle Li, Yanan Hua, Caixia Yang, Sijing Liu, Li Tan, Jinlin Guo, Yang Li

**Affiliations:** 1 Key Laboratory of Characteristic Chinese Medicine Resources in Southwest China, College of Pharmacy, Chengdu University of Traditional Chinese Medicine, Chengdu, P. R. China; 2 College of Medical Technology, Chengdu University of Traditional Chinese Medicine, Chengdu, P. R. China; 3 School of Public Health, Chengdu University of Traditional Chinese Medicine, Chengdu, P. R. China

**Keywords:** *Mori Fructus*, Polysaccharide, Oxidative stress, PI3K/AKT-Nrf2 signaling pathway

## Abstract

**Background:**

*Mori Fructus* is an economical and readily available traditional Chinese medicine and food. Polysaccharides in *Mori Fructus* have clear antioxidant activity and have been found to alleviate oxidative stress (OS)-induced liver damage in experimental studies. The mechanism of regulation of cellular antioxidant activity by mulberry polysaccharides has been suggested to be Nrf2, but it is not clear whether the Nrf2 pathway is mediated by activation of other targets, and the exact process of effects in hepatocytes has yet to be elucidated.

**Methods:**

In this study, the basic characterization of total polysaccharides extracted from mulberry fruits (Morus nigra *Linn.*) was analyzed. A model of oxidative damage induced by H_2_O_2_ in HepG2 cells was established. The levels of cellular oxidation-related markers, including ROS, SOD and Gpx, were then examined. Furthermore, Q-PCR and Western-blot were used to detect the expression of genes and proteins related to the PI3K/Akt-mediated Nrf2 signaling pathway.

**Results:**

The results showed that a total mulberry polysaccharides (TMP) has a molecular weight of 57.5 kDa with a pyranose ring mainly composed of glucose (48.81%), galactose (22.79%) and mannose (18.2%). TMP reduced the accumulation of ROS in HepG2 cells after H_2_O_2_ treatment and modulated the activity of SOD and Gpx. Q-PCR and Western-blot showed that TMP could up-regulate the expression of p-PI3K, p-AKT, Nrf2, NQO1 and HO-1.

**Conclusions:**

This study demonstrates that TMP can reduce ROS accumulation in H_2_O_2_-treated HepG2 cells and restore cell viability by activating the PI3K/AKT-mediated Nrf2 pathway. TMP may be a potent antioxidant agent that could slow down oxidative damage to the liver.

**Supplementary Information:**

The online version contains supplementary material available at 10.1186/s12906-023-03925-w.

## Background

*Mori Fructus*, also called mulberry, is the fruiting spike of the mulberry tree (*Morus alba* L.) of the mulberry family, which is widely distributed, native to India and China at the foot of the Himalayas, now widely distributing in tropical and temperate zones [1]. The common mulberry species are red mulberry (*Morus rubra* Linn.), black mulberry (*Morus nigra* Linn.) and white mulberry (*Morus alba* Linn.) [2]. It is worth noting that it is widely used not only in the food industry but also as a traditional Chinese medicine [3].

Plant polysaccharides are widely in leaves, fruits and flowers with active property [4]. Its antioxidant properties are the focus of more and more scholarly attention after polyphenols, flavonoids and tannins [5]. Polysaccharides is the main active ingredient in mulberries [6], which is a kind of pectin-type polysaccharide extracted and purified from mulberries [7]. Numerous studies have shown that mulberry polysaccharides have strong antioxidant properties in vivo and in vitro, and that there is a correlation with structure and activity [8–13]. Further, studies have found that the good antioxidant properties of mulberry polysaccharides have an anti-liver damage effect [14, 15].

Liver disease is one of the most frequent diseases in the world and liver injury is closely related to it [16]. Oxidative stress (OS) is an important pathogenesis of chronic liver injury [17]. OS is caused by excessive reactive oxygen species (ROS). However, ROS at normal levels can maintain normal physiological functions in cells, when ROS is excessive it damages proteins, lipids and DNA [18]. It is an important pathogenesis of liver injury in liver fibrosis, non-alcoholic fatty liver disease, alcoholic liver disease and viral hepatitis [19]. Live cells are susceptible to exogenous or endogenous highly oxidizing molecules. The highly oxidizing molecule H_2_O_2_ is commonly used as a reagent to simulate oxidative damage in antioxidant capacity/mechanism experiments [20, 21].

Notably, plant polysaccharides can develop their antioxidant activity by scavenging free radicals, improving antioxidant enzyme activity or modulating antioxidant signaling pathways [22, 23]. Currently, researchers have found that mulberry polysaccharides can alleviate oxidative damage by scavenging free radicals, enhancing antioxidant enzyme activity and regulating the Nrf2 pathway, which is closely related to OS [24, 25]. Whereas polysaccharides pass through a variety of antioxidant signaling pathways, researches on the modulation of antioxidant signaling pathways in mulberry polysaccharides have focused on the downstream regulation of Nrf2 [22, 25–27]. Further, the PI3K/AKT pathway can regulate the development of the Nrf2 signaling pathway [28, 29]. It has been demonstrated that polysaccharides from Morchella esculenta can stimulate the PI3K/AKT-mediated Nrf2 pathway to reduce oxidative damage in human alveolar epithelial A549 cells [30]. However, it is not clear whether mulberry polysaccharides can also regulate the PI3K/AKT-mediated Nrf2 pathway in HepG2 cells.

In order to examine the above hypothesis, a total mulberry polysaccharides (TMP) was extracted from mulberry fruits (*Morus nigra* Linn.) and its basic characteristics were described in this study. Using H_2_O_2_ as an inducer of OS, the effects of TMP on ROS levels and antioxidant enzyme activities in oxidatively injured hepatocytes were analyzed, focusing on the regulation of PI3K/AKT-mediated Nrf2 pathway by TMP.

## Materials and Methods

### Materials and reagents

The *Mori Fructus* fruits (*Morus nigra* Linn.) samples were sourced from Guangxi Province (China), 30% hydrogen peroxide was bought from Chengdu Keen (Chengdu, China), ROS, SOD, Gpx, BCA protein quantification and plasmatic nucleus isolation protein extraction kits are all from Beyotime Institute of Biotechnology (Shanghai, China). Cell Counting Kit-8(CCK-8) bought from Everbright, MEM medium, phosphate buffer and NQO1 antibodies were obtained from Boster (California, America). Lamin B and Nrf2 antibodies were from Proteintech (Wuhan, China), HO-1, Gapdh, AKT, p-AKT p-PI3K and PI3K antibodies were all obtained from Affinity(America), goat anti-mouse from Beyotime and goat anti-rabbit from Affinity.

### Preparation of TMP

According to the response surface method [31], weigh 6 g of mulberry fruits (*Morus nigra* Linn.) powder, add 96ml of ultrapure water to obtain the material-to-liquid ratio of 1:16, ultrasonic extraction for 22 min at 220w and 70 ℃. Centrifuge at 5000×g for 10 min, collect the supernatant, add anhydrous ethanol to 80% ethanol (v/v), then stewing at 4 ℃ overnight, and discard the supernatant. After redissolving the precipitate in distilled water, it was deproteinated 5 times with Sevag reagent (chloroform: n-butyl alcohol = 5:1, v/v) until the middle layer was free of white flocculence. Dialyze the crude polysaccharide for 48 h, change the dialysate every 12 h to remove the organic matter. Finally, the TMP was obtained after freeze-drying.

### Chemical composition and Molecular Weight determination of TMP

Phenol-sulfuric acid method was used to analyze the content of total polysaccharides in TMP. A UV spectrophotometer (200–600 nm, Mipta UV-3100PC, China) was used to detect the protein content. The molecular weight and homogeneity of TMP were determined by a high-performance gel permeation chromatography (HPGPC) (Shimadzu, GPC-20 A, Japan) coupled with a differential refractive index detector-20 (RID-20 A) and a gel chromatography column (Waters No. WAT011525). 20µL of TMP solution (2.5 mg/mL) was injected to analyze. The flow rate was set at 1 mL/min, the column temperature at 35 °C and the detector temperature at 40 ℃. Glucose (T26300, T12600, T5800, T4290, T1400, T1030, T633, T342, T180, T150) was used as the standard.

### Fourier Transform Infrared (FT-IR) spectrometry analysis of TMP

5 mg of dried TMP was made into a thin film together with KBr [32] and measured at 400–4000 cm^− 1^ under FT-IR instrument (Thermo Nicolet Corporation, iS10, USA) with a spectrometer resolution of 4 cm^− 1^ and 32 scans.

### Monosaccharide Composition Analysis of TMP

10 mg of TMP was hydrolyzed with 2 mol/L at TFA, 120 ℃ for 4 h to obtain monosaccharides. Then the solution was analyzed by HPLC (Shimadzu LC-20AD, column: Xtimate C18 4.6*200 mm 5 μm) at 250 nm for the monosaccharide composition. Mannose (Man), ribose (Rib), rhamnose (Rha), glucuronide (GlcUA), galacturonide (GalA), N-acetyl-glucosamine (GlcNAc), glucose (Glu), N-acetyl-aminogalactose (GalNAc), galactose (Gal), xylose (Xyl), arabinose (Ara) and fucose (Fuc) were used as standards for the quantitative and qualitative analysis of monosaccharides.

### HepG2 cell lines and culture

HepG2 cells were obtained from Shanghai Cell Bank, Chinese Academy of Sciences. HepG2 cells was incubated in MEM medium (BOSTER, China) containing 10% fetal calf serum (Sorfa, China) with 1% streptomycin (Biosharp), 5% CO_2_, 37 °C incubator.

### TMP cytotoxicity assay

The effect of TMP on the viability of HepG2 cells was determined by the CCK-8 method, in which WST-8 in the reagent can generate yellow methanogenic products in the presence of dehydrogenase. In a 96-well plate, 100 µL of each well was seeded with a density of 1 × 10^5^ HepG2 cells and incubated in the incubator for 24 h. After incubation, different concentrations of TMP (0.05, 0.1 and 0.2 mg/mL) were added and incubation was continued for 24 h [26]. Finally, each well was incubated for 30 min in the incubator by adding 10% CCK-8 reagent, and the absorbance of each group was detected at 450 nm.

### Oxidative damage concentration of HepG2 cells Induced by H_2_O_2_

Each well was seeded with 100 µL at a density of 1 × 10^5^ HepG2 cells in a 96-well plate. After incubation for 24 h, different concentrations of H_2_O_2_ (300, 600, 900 and 1200 µmol/L) were added and incubated for another 2 h [25]. Finally, the absorbance of HepG2 cells was measured at 490 nm by the CCK-8 method to screen for the optimal concentration of H_2_O_2_ causing oxidative damage to the cells. In which, no H_2_O_2_ was added for the normal group of cells.

### Protective Effects of TMP on the HepG2 in the Condition of H_2_O_2_ oxidative damage

HepG2 cells were planted in 96-well plates with 100 µL at a density of 1 × 10^5^/well, added different concentrations of TMP (0.05, 0.1 and 0.2 mg/mL) in the incubator for 24 h and then incubated with the optimal concentration of H_2_O_2_ for 2 h [25]. The viability of HepG2 cells was determined by the CCK-8 method to reflect the protective effects of TMP.

### ROS Assay

HepG2 cells were grown in 6-well plates at 8 × 10^5^/well and different concentrations of TMP were added after one day. After 24 h, H_2_O_2_ was added at the optimal concentration. ROS content was determined according to the kit instructions (Beyotime, China) [33], and the fluorescence values of each group were measured using a fluorescence spectrometer (F4600, Hitachi).

### SOD and gpx activity assay

SOD and Gpx activities were determined according to the kit instructions (Beyotime, China). SOD activity was analyzed by the WST-8 method, which produces a methanogenic dye that is inversely proportional to the activity of SOD in the catalytic reaction with xanthine oxidase [34]. Gpx activity was measured by the DNTB method, which measures the activity of Gpx based on the yellow TNB formed by the reaction of the remaining GSH. SOD and Gpx activity may be reduced after oxidative damage to cells.

### Quantitative real-time PCR (Q-PCR)

HepG2 cells were grown in 6-well plates at 8 × 10^5^/well and different concentrations of TMP (0.05 and 0.1 mg/ml) were added after one day. After 24 h, 600 µmol/L of H_2_O_2_ was added for 2 h. And total RNA was extracted with the kits (Biomarker, China). After that, 800ng of RNA was reverse transcribed to cDNA using the BiomarkerScript III RT Master Mix for qPCR kit (Biomarker, China) according to the instructions. The denaturation was carried out at 95 °C for 3 min, followed by 40 cycles at 90 °C for 5 s and 60 °C for 30s. Finally GAPDH was used for normalization with 2^−ΔΔCT^ values to calculate relative mRNA expression. The primer design of this experiment is shown in Table 1 [25].

**Table 1:** Primer sequences for Q-PCR

*Primers*	*Sequences*
NQO1	F,CTGATCGTACTGGCTCACTC;R,GAACAGACTCGGCAGGATAC
HO-1	F,CCAGGCAGAGAATGCTGAGT;R,GTAGACAGGGGCGAAGACTG
GAPDH	F,GGAGCGAGATCCCTCCAAAAT;R,GGCTGTTGTCATACTTCCTCATGG

### Western blot analysis

HepG2 cells were cultured in line with the above. Nuclear and total cellular proteins were extracted using commercial kits (Beyotime, China). Protein concentrations were also determined using an enhanced BCA protein kit (Beyotime, China). Cellular protein samples were separated by sodium dodecyl sulfate (SDS)-polyacrylamide gel electrophoresis and then electrotransferred to polyvinylidene fluoride (PVDF) membranes. Blocking was performed for 40 min using Protein Free Rapid Blocking Buffer (Yamei, China), and primary antibody blocking was performed overnight at 4 °C. The membrane was then washed three times with T-TBST for 10 min each and then bound to the secondary antibody for 1 h at room temperature. After three more T-TBST washes, the kit was detected using an ECL kit with an automated chemiluminescence image analysis system (BIO-RAD, America). The western blots were analyzed using ImageJ software (NIH, Bethesda, MD, USA). And the blots were cut prior to hybridisation with antibodies during blotting.

### Statistics Analysis

The results are expressed as mean ± standard deviation from three independent experiments, with differences among groups compared using GraphPad Prism 9.0 software with One-way ANOVA and P < 0.05 was considered a statistically significant difference.

## Results and discussion

### The characterization of TMP

The extraction yield is 1.71 g/100 g. The chemical composition of the TMP is listed in Table 2.

**Table 2:** Physicochemical analysis of TMP

Parameter	Composition,%
Total polysaccharides	76.7 ± 1.46
Protein content	2.01 ± 1.01

The molecular weight of TMP was determined by HPGPC, and the regression equation was y=-1.5507x + 13.691 (x is retention time and y is LogMW). The average molecular weight of the TMP was 57.5 KDa. The polydispersity (Mz/Mw) of the TMP was 1.009, and the closer the Mz/Mw was to 1 indicated the more concentrated the molecular weight distribution of the TMP.

**Fig. 1:**
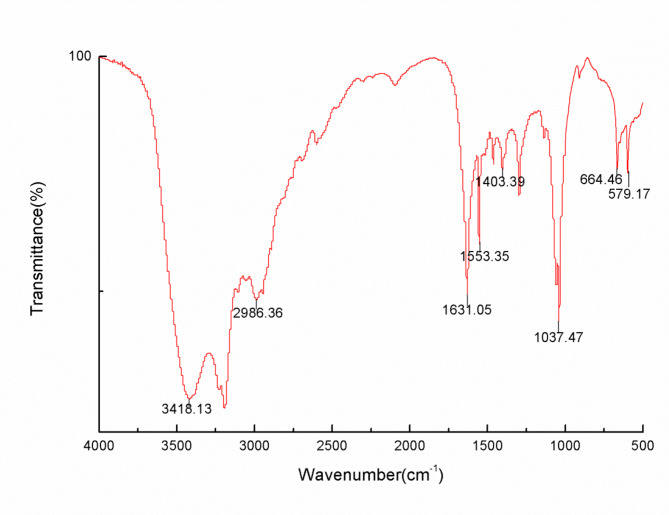
FT-IR spectra of TMP

The FT-IR spectrogram of TMP used for the analysis of characteristic absorption peaks of polysaccharides is shown in Fig. 1. The broad band around 3400 cm^− 1^ in the spectrum is the stretching vibration of O-H, while the absorption near 2986 cm^− 1^ is due to stretching and bending vibrations of C-H [35, 36]. The strong vibrational peaks at 1631 cm^− 1^ and 1460 cm^− 1^ are vibrations of the C = O bond, suggesting the presence of carboxyl groups [37]. And around 1037 cm^− 1^ is a typical vibration of glycosidic bond C = O = C caused by the stretching vibration of the pyran ring probably [31]. The FT-IR spectral results suggest that TMP may be a polysaccharide with a pyranic ring.

**Fig. 2:**
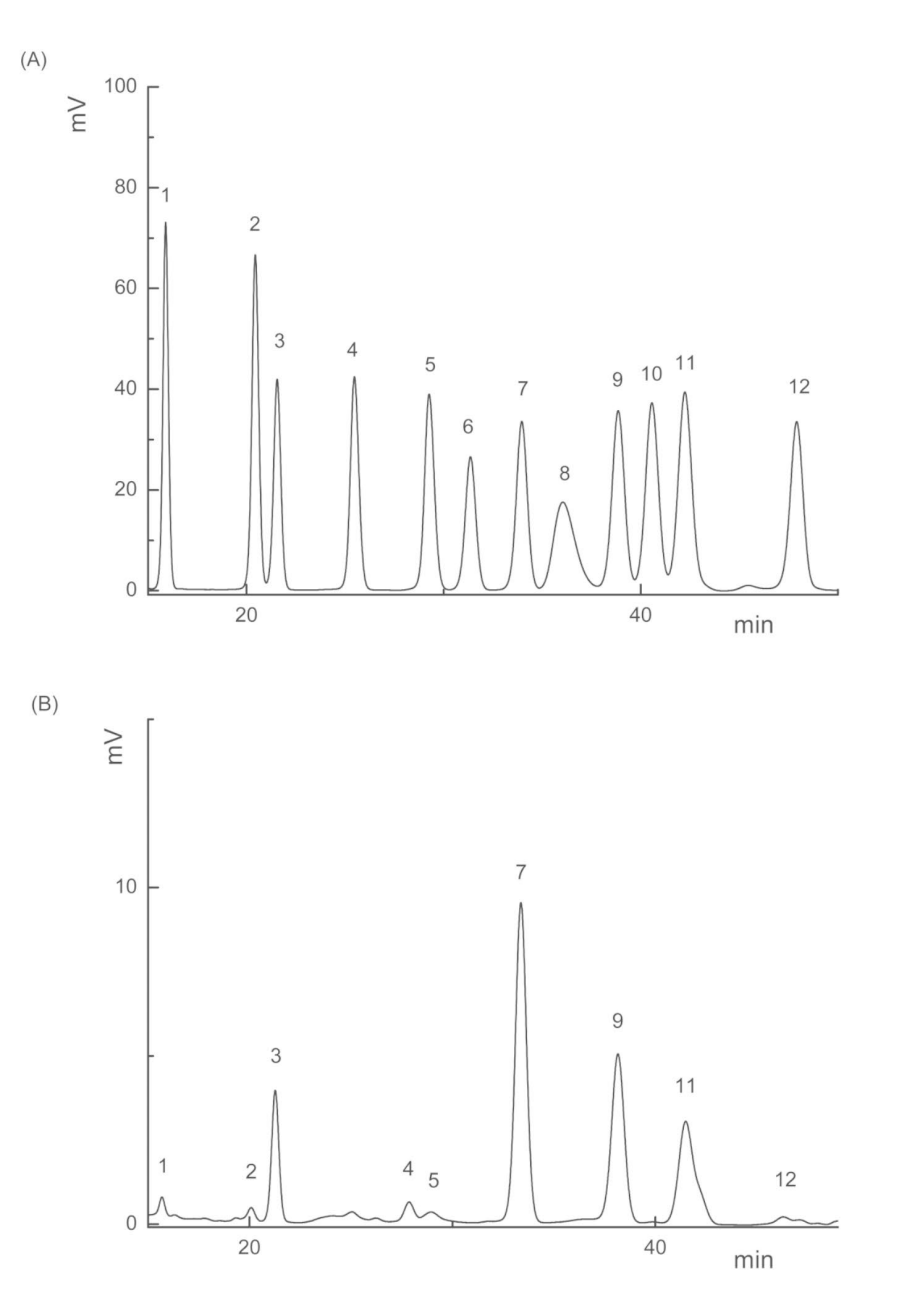
HPLC analysis of standard monosaccharides **(A)** and TMP **(B)**. (1 Man, 2 Rib, 3 Rha, 4 GlcUA, 5 GalA, 6 GlcNAc, 7 Glu, 8 GalNAc, 9 Gal, 10 Xyl, 11 Ara, 12 Fuc)

The monosaccharide composition of TMP was analyzed using HPLC system (Fig. 2). The results show that TMP consists mainly of glucose (48.81%), galactose (22.79%) and mannose (18.2%), but contains small amounts of ribose (0.43%), rhamnose (0.082%), glucuronide (1.56%), galacturonic acid (0.041%), arabinose (1%), and fucose (0.22%). While the monosaccharide composition of TMP is different from the polysaccharide of the mulberry harvested in the provinces of Zhejiang and Guizhou [25, 38]. Probably intra-species variation due to different origins [39].

### Effects of TMP on H_2_O_2_-Induce OS

**Fig. 3:**
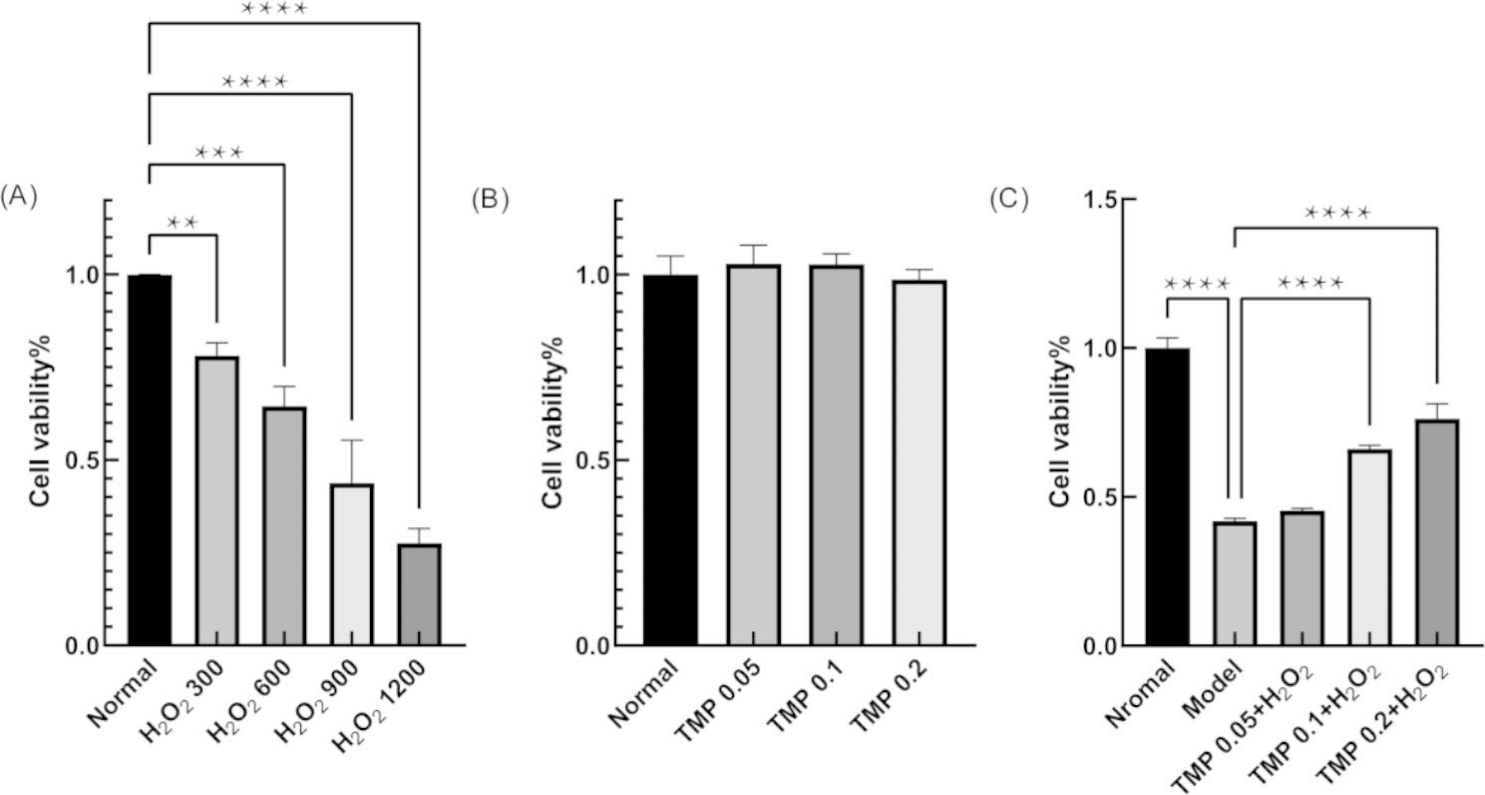
The effects of TMP on HepG2 by CCK-8 assay. **(A)** Cell viability results of HepG2 cells treated with different concentrations of H_2_O_2_ (300, 600, 900 and 1200 µmol/L) **(B)** Effects of different concentrations of TMP (0.05, 0.1 and 0.2 mg/mL) on the viability of HepG2 cells **(C)** Protective effect of TMP on HepG2 cells in the oxidation damage of H_2_O_2_. (*P < 0.05, **P < 0.01, ***P < 0.001 and ****P < 0.0001)

It was not until the 1950s that the toxicity of hydrogen peroxide showed its true significance in biology [20]. One of the side effects of many oxidative enzymes that catalyze the metabolism of amino acids, purines and fatty acids is hydrogen peroxide. Numerous studies have shown that different concentrations of H_2_O_2_ acting on cells can cause different degrees of oxidative damage to cells [21, 40]. First, find a suitable H_2_O_2_ concentration. After treating HepG2 cells with different concentrations of H_2_O_2_ (300, 600, 900 and 1200 µmol/L) for 2 h, we observed that HepG2 cells morphology was damaged. And the results showed that the viability of HepG2 cells decreased (*P*＜0.01, 0.001 and 0.0001) with the increase of H_2_O_2_ concentration (Fig. 3A). When the H_2_O_2_ concentration was 600 µmol/L, the cell viability was about 50%, so this concentration was chosen for subsequent experiments.

To investigate whether TMP itself has any toxicity on the survival of HepG2 cells, HepG2 cells were treated with 0.05, 0.1 and 0.2 mg/mL of TMP for 24 h. The CCK-8 results (Fig. 3B) showed the cell proliferation rate remained at around 100% in the presence of TMP, suggesting that TMP (0.05, 0.1, and 0.2 mg/mL) is not found to be toxic to the growth of HepG2 cells.

To further understand the effect of TMP on HepG2 cells induced by H_2_O_2_. HepG2 cells were pretreated with 0.05, 0.1, and 0.2 mg/mL of TMP for 24 h, and later treated with 600 µmol/L of H_2_O_2_ for 2 h. The results showed that the survival rate of HepG2 cells pre-protected with TMP increased dose-dependently (*P*＜0.0001) with the TMP concentration (Fig. 3C). This result indicates that TMP has a significant protective effect on HepG2 cells with H_2_O_2_-induced oxidative damage.

### TMP inhibits ROS Accumulation

**Fig. 4:**
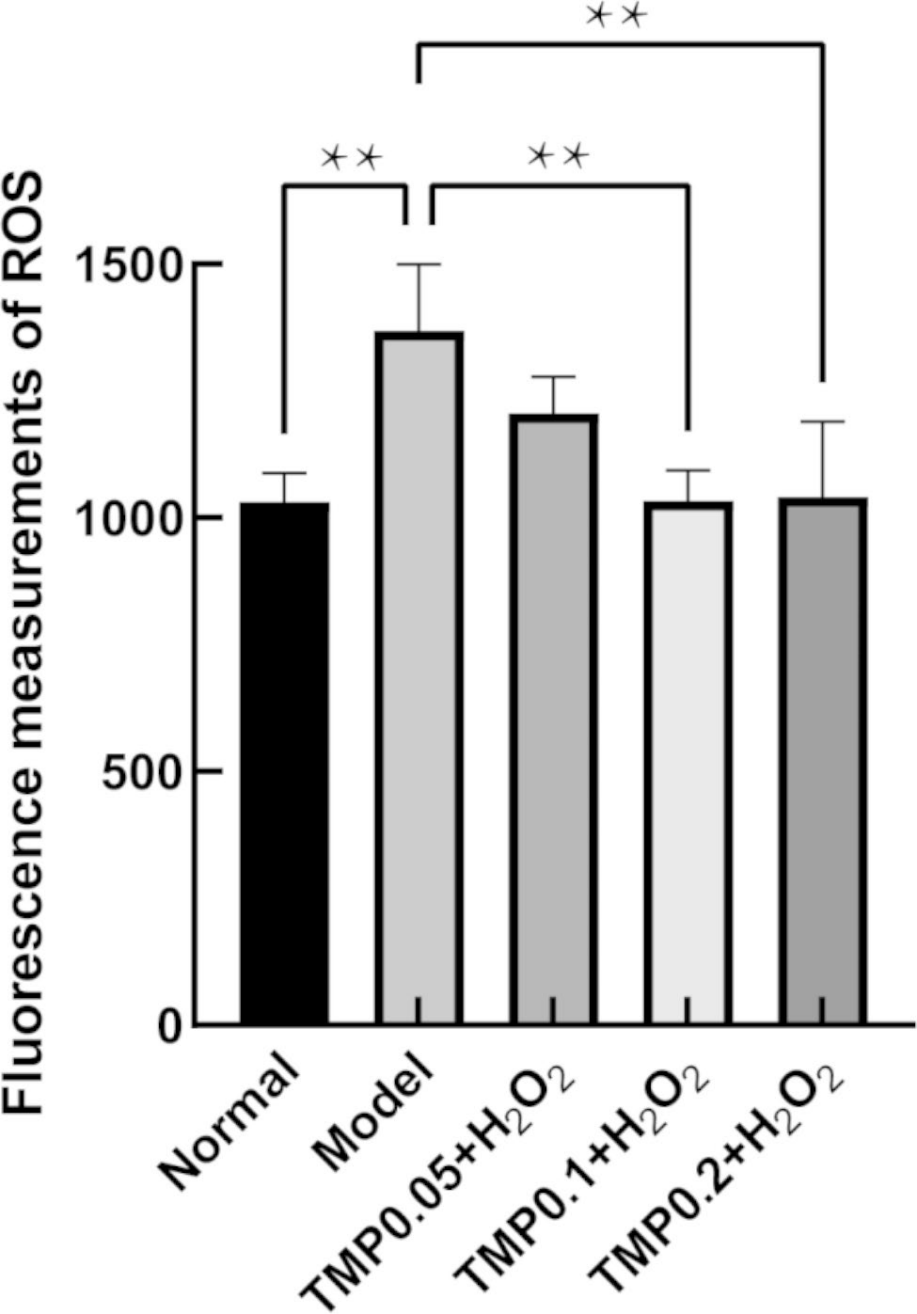
TMP effects on the H_2_O_2_-induced ROS accumulation in HepG2 cells. (**P < 0.01)

ROS plays a key role in OS. If the concentration of ROS is high, it will react with DNA and proteins, etc., causing an imbalance between the antioxidant and oxidative systems, which eventually leads to OS and seriously affects cell survival [39, 41–43]. The ROS content in the normal, model and treated groups was demonstrated in Fig. 4. The fluorescence values were significantly higher in the model group compared with the normal group, indicating that the model group had higher intracellular ROS levels and OS had been caused. However, the Fluorescence values in the TMP-treated group were significantly lower, suggesting a reduction in ROS accumulation (*P*＜0.01). The ROS was reduced to the same level of the normal group as the TMP concentration reached 0.1 mg/mL. The ROS results suggest that TMP can inhibit the accumulation of the ROS in by H_2_O_2_-induced HepG2 cells, which plays a role in alleviating oxidative damage.

### TMP improved the SOD and GPX activities

**Fig. 5:**
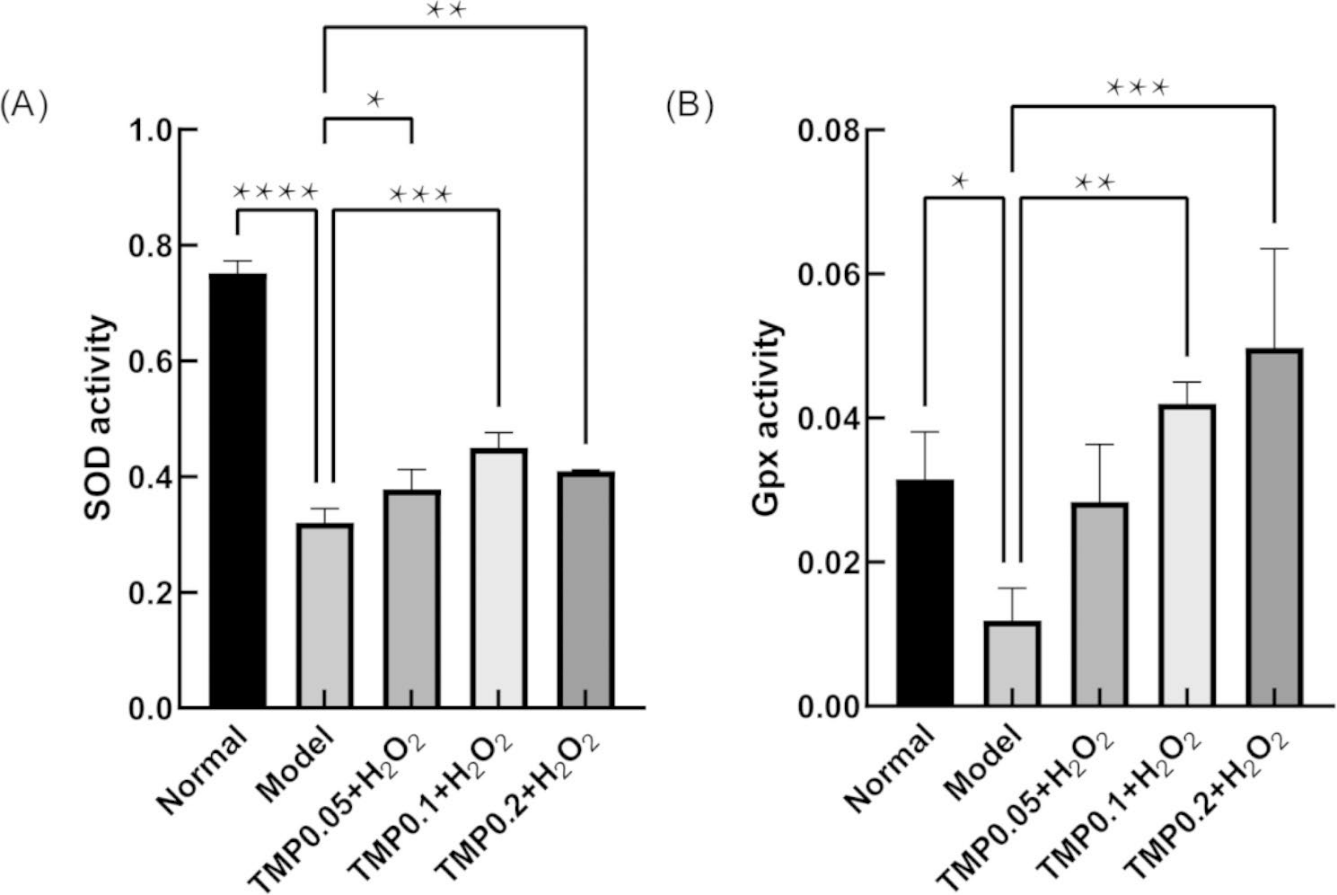
Effects of TMP on antioxidant enzyme activities changes in HepG2 cells. **(A)** Changes in SOD activity after TMP (0.05, 0.1 and 0.2 mg/mL) pre-protection. **(B)** Changes in Gpx activity after TMP pre-protection (0.05, 0.1 and 0.2 mg/mL). (*P < 0.05, **P < 0.01, ***P < 0.001 and ****P < 0.0001)

The antioxidant enzymes SOD and Gpx can act synergistically with proteins such as peroxide dismutase, thioredoxin (Trx) and glutathione (Grx) and some small molecular weight antioxidants to scavenge ROS [20, 42, 44]. So we measured the activities of SOD and Gpx in this experiment. The results showed (Fig. 5) that the activities of SOD and Gpx were significantly lower in the model group compared with the normal group, indicating that the intracellular oxidative homeostasis had been disrupted. In the treated group (TMP 0.1 mg/ml and TPM 0.2 mg/ml), the activities of SOD and Gpx were significantly raised (*P*＜0.01 and 0.001), and the opposite trend to the ROS results, indicating that TMP improved the activities of SOD and Gpx to scavenge the excess ROS produced by H_2_O_2_-induced OS in HepG2 cells. It is worth mentioning that the results of this experiment were not consistent with the results of Mulberry in Zhejiang [25, 26].

### TMP activated the PI3K/AKT-Mediated Nrf2 signaling pathway

**Fig. 6:**
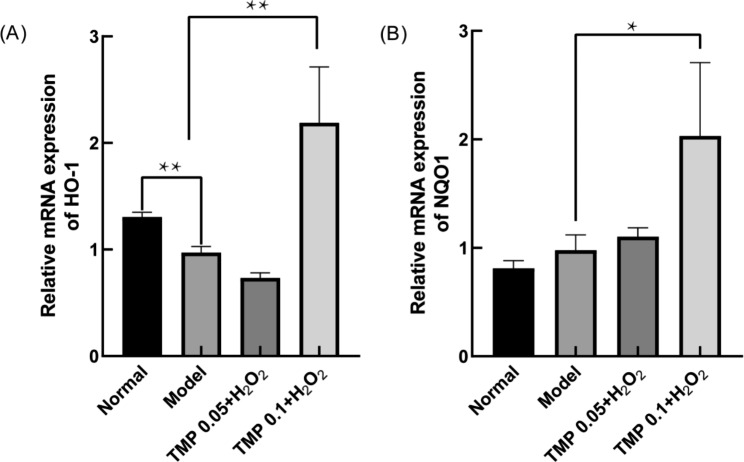
Effects of TMP on mRNA changes in HepG2 cells. **(A)** Changes in HO-1 mRNA after TMP (0.05, and 0.1 mg/mL) pre-protection. **(B)** Changes in NQO1 mRNA after TMP (0.05, and 0.1 mg/mL) pre-protection. (*P < 0.05, **P < 0.01)

NF-E2-related factor 2(Nrf2) is a transcription factor usually tightly bound to Keap1 in the cytoplasm [44]. When the active cysteine amino acids of Keap1 are modified, Nrf2 is released by Keap1 [45] and enters the nucleus to interact with ARE sequences to initiate the expression of antioxidant (SOD, Gpx) as well as downstream related genes such as HO-1 and NQO1 to remove excess ROS [46, 47]. PI3K activates AKT to regulate cell proliferation, differentiation and apoptosis [44, 48], while PI3K/AKT is an upstream regulator of Nrf2, a major antioxidant response agent [46]. To investigate the mechanism of TMP on H_2_O_2_-induced oxidative damage in HepG2 cells, we first analyzed the changes of mRNA expression of Genes downstream of Nrf2, the results showed (Fig. 6) that TMP at 0.1 mg/ml significantly increased (*P*＜0.05 and 0.01) the expression of HO-1 and NQO1. Several studies have shown that when OS occurs in mammalian cells, protein expression patterns may be regulated [49].

**Fig. 7:**
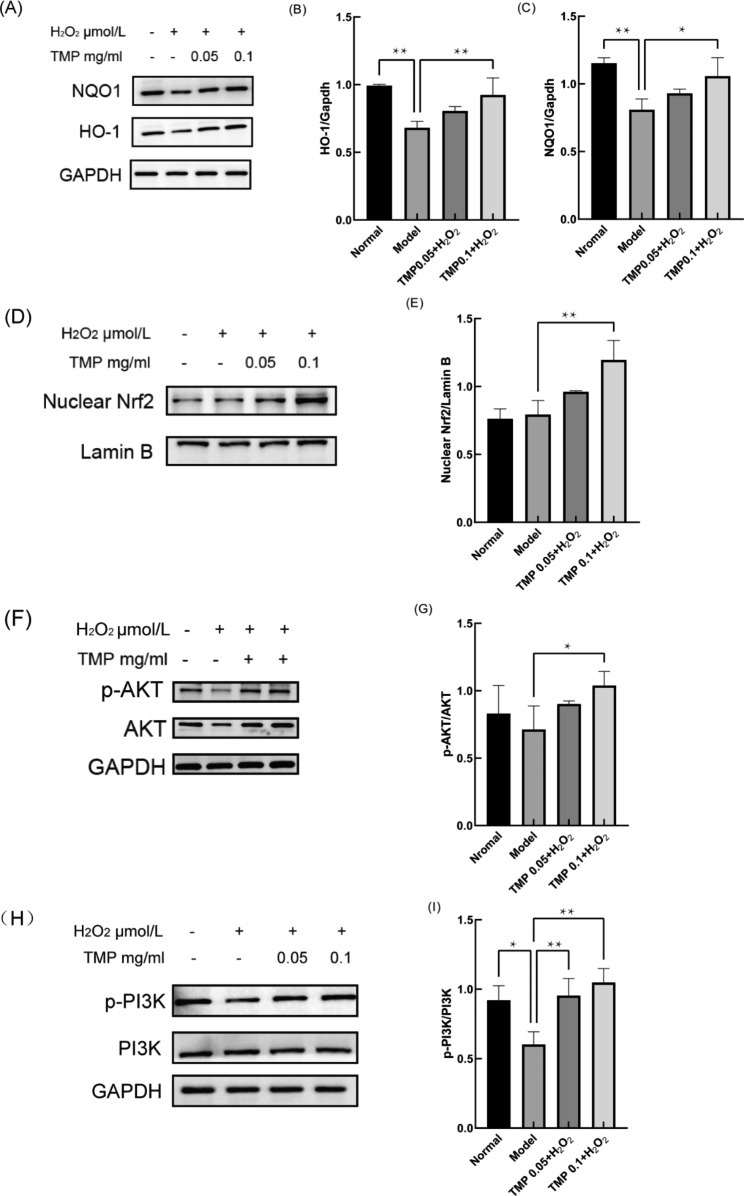
Effects of TMP on PI3K/AKT/Nrf2 signaling pathway in HepG2 cells. **(A-C)** Effects of TMP (0.05, and 0.1 mg/mL) on HO-1 and NQO1 proteins Changes in HepG2 cells. **(D-E)** Effects of TMP (0.05, and 0.1 mg/mL) on nuclear Nrf2 protein Changes in HepG2 cells. **(F-I)** Effects of TMP (0.05, and 0.1 mg/mL) on p-AKT and p-PI3K proteins Changes in HepG2 cells. (*P < 0.05, **P < 0.01)

To further investigate the protective mechanism of TMP against H_2_O_2_-induced liver injury, we investigated the expression changes of related antioxidant proteins. The outcomes of Western Blot showed (Fig. 7) that the expression of key nuclear protein Nrf2 content increased in the treated group (*P*＜0.01), suggesting that TMP plays a role in promoting the entry of Nrf2 into the nucleus. To investigate whether TMP activates Nrf2 via PI3K/AKT, we examined the expression of p-PI3K and p-AKT proteins. And the increasing of p-PI3K and p-AKT after TMP pre-protection (*P*＜0.05 and 0.01) indicated that the intra nuclear aggregation of Nrf2 was accelerated through the PI3K/AKT pathway. Meanwhile the downstream proteins NQO1 and HO-1 of Nrf2 also increased (*P*＜0.05 and 0.01). Altogether, we found that the mRNA levels of antioxidant genes HO-1 and NQO1 and PI3K/AKT-mediated Nrf2 pathway-related proteins were both significantly upregulated after TMP pretreatment. These results suggest that TMP may activate the PI3K/AKT-mediated Nrf2 pathway and alleviate H_2_O_2_-induced liver injury in HepG2 cells.

**Fig. 8:**
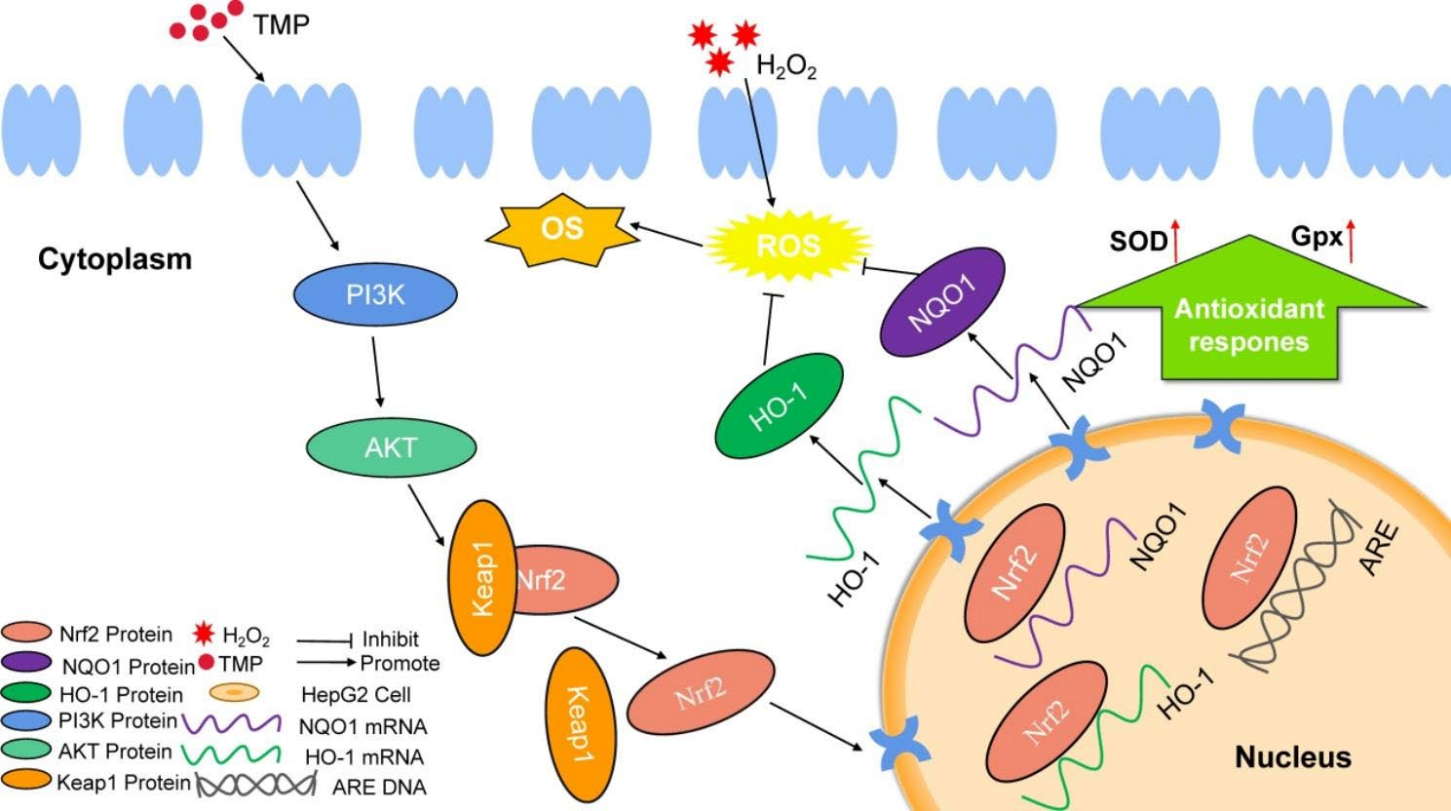
A summary diagram. TMP protects against H_2_O_2_-induced liver injury via PI3K/AKT-mediated Nrf2 pathway

As shown in Fig. 8, TMP may inhibit the accumulation of ROS in cells by stimulating the phosphorylation of PI3K and AKT to accelerate the entry of Nrf2 into the nucleus, thereby stimulating the expression of OH-1 and NQO1 downstream of Nrf2 and upregulating the activity of SOD and Gpx. The antioxidant activity of TMP was thus shown.

## Conclusion

In this study, TMP was obtained from mulberry fruits (*Morus nigra* Linn.) and provided evidence that TMP eased H_2_O_2_-induced oxidative damage in HepG2 cells. We report polysaccharides from mulberry fruits with a pyranose ring mainly composed of glucose (48.81%), galactose (22.79%) and mannose (18.82%) whose molecular weight is 57.5 kDa. The hepatoprotective effect of TMP on H_2_O_2_-induced hepatic injury in HepG2 cells may be through upregulation of SOD and Gpx with activation of PI3K/AKT-mediated Nrf2 pathway to alleviate OS. This study provides new ideas for the development of mulberry polysaccharides as an antioxidant regent, protecting the liver against OS. As a continuation, at least the following two aspects are worth further study. One is the key domain of TMP in the activation of antioxidant signaling pathway in hepatocytes. Another is receptors on the surface of hepatocytes receiving TMP stimulation signals and their correlation with antioxidant signaling pathway.

## Electronic supplementary material

Below is the link to the electronic supplementary material.

**Additional file 1**: Supplementary Information

## Data Availability

The datasets generated during and analyzed during the current study are available from the corresponding author on reasonable request.

## Abbreviations

FI-IR: Fourier transform infrared spectroscopy
HPGPC: High performance gel permeation chromatography
HPLC: High performance liquid chromatography
Nrf2: NF-E2-related factor 2
OS: Oxidative stress
Q-PCR: Quantitative real-time PCR
TMP: A total mulberry polysaccharide
