# Supplementary material for: Polysaccharides extracted from mulberry fruits (Morus nigra L.): antioxidant effect of ameliorating H2O2-induced liver injury in HepG2 cells
Source: BMC Complement Med Ther. 2023 Apr 12;23:112. doi: 10.1186/s12906-023-03925-w (PMC10091537; doi:10.1186/s12906-023-03925-w)

Supplementary Information

Journal: BMC Complementary Medicine and Therapies

Title: "Polysaccharides extracted from mulberry fruits (*Morus nigra* L.): antioxidant effect of ameliorating H_2O_2-induced liver injury in HepG2 cells"

Xinle Li[1*], Yanan Hua[3*], Caixia Yang[1], Sijing Liu [3], Li Tan[1], Jinlin Guo[1,3#], Yang Li[2#]

[1] Key Laboratory of Characteristic Chinese Medicine Resources in Southwest China, College of Pharmacy, Chengdu University of Traditional Chinese Medicine, Chengdu, P. R. China. [2] School of Public Health, Chengdu University of Traditional Chinese Medicine, Chengdu, P. R. China.[3] College of Medical Technology, Chengdu University of Traditional Chinese Medicine, Chengdu, P. R. China;

[#]Correspondence: liyang@cdutcm.edu.cn (Y. L.) and guo596@cdutcm.edu.cn (J. G.)

The original image of the protein blots are shown below (Including all repeated images), and the protein blot results in A are from the same experiment. (Same for B, C, D) The results of gel blotting experiments are circled in red, samples not circled are from other experiments and are used to prevent edge effects.

(A) Western blot original strips of NQO1, HO-1, GAPDH (Including all repeated images).

NQO1 31kDa

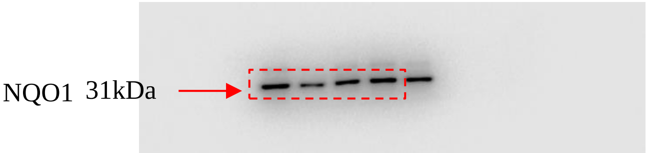

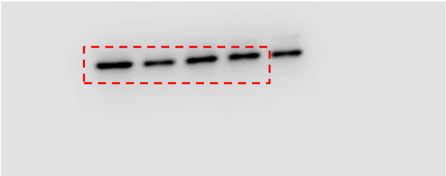

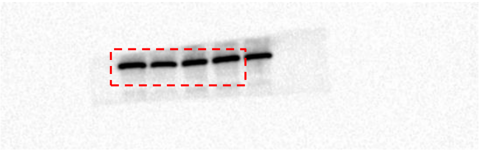

HO-1 33kDa

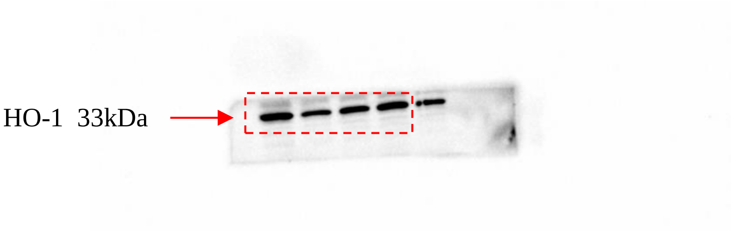

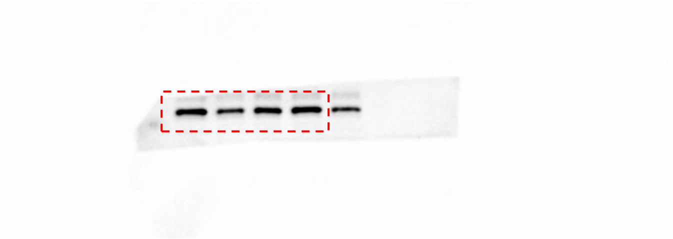

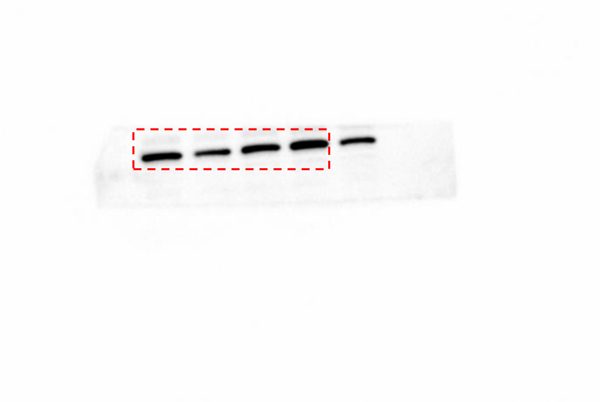

GAPDH 36kDa

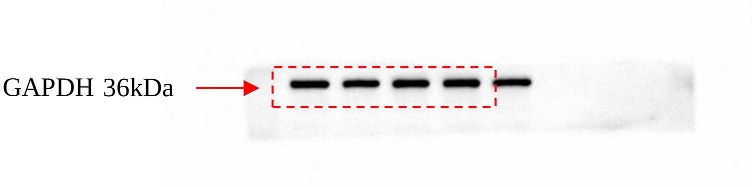

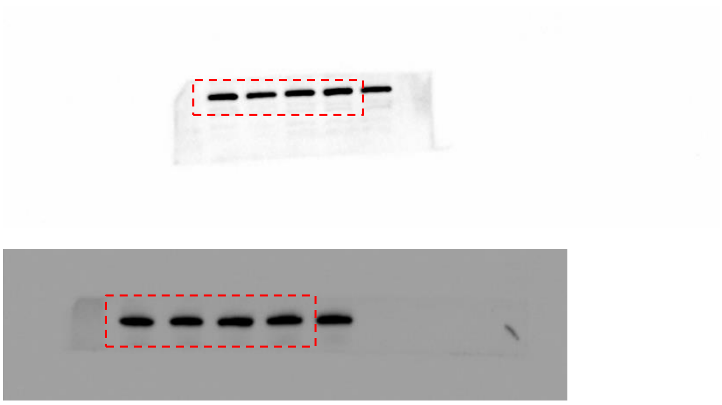

(B) Western blot original strips of Nrf2 and Lamin B (Including all repeated images).

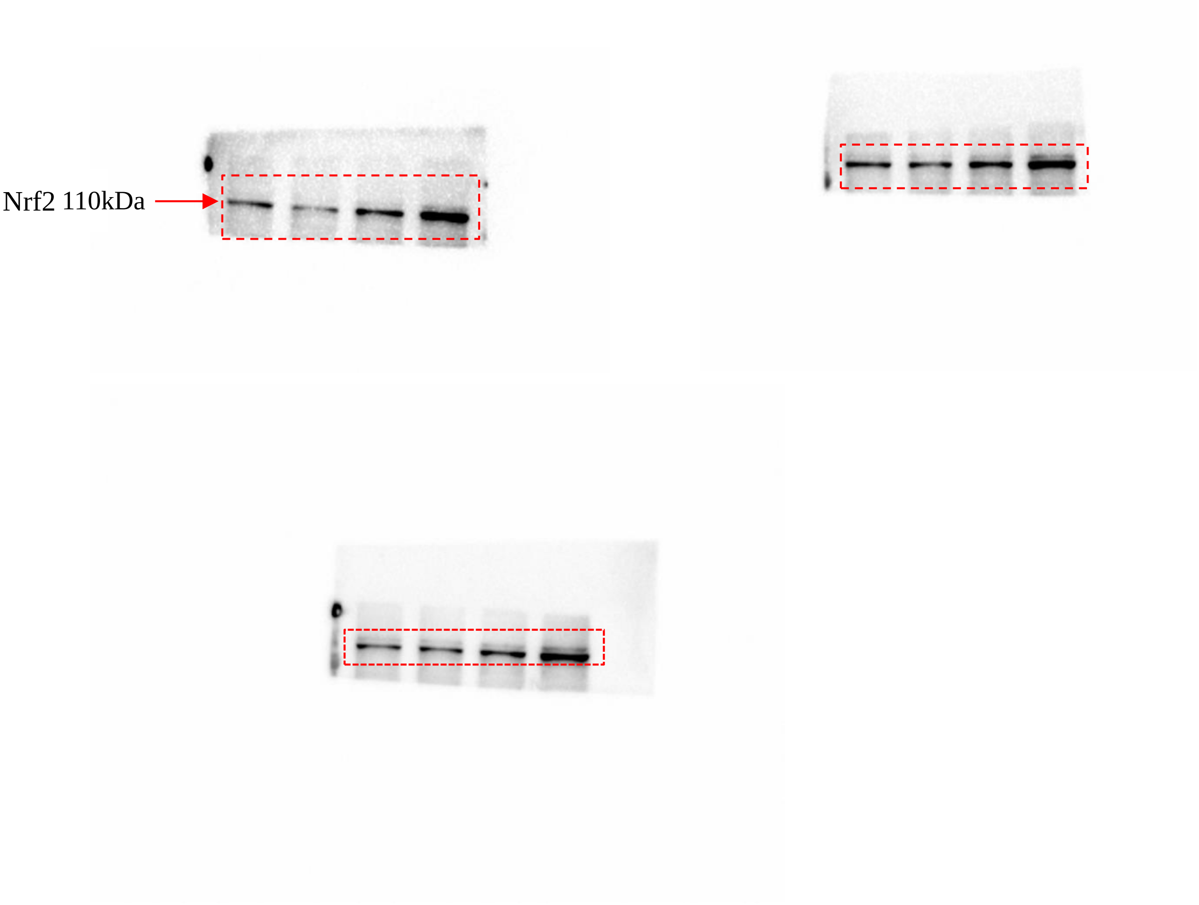

Lamin B 66kDa

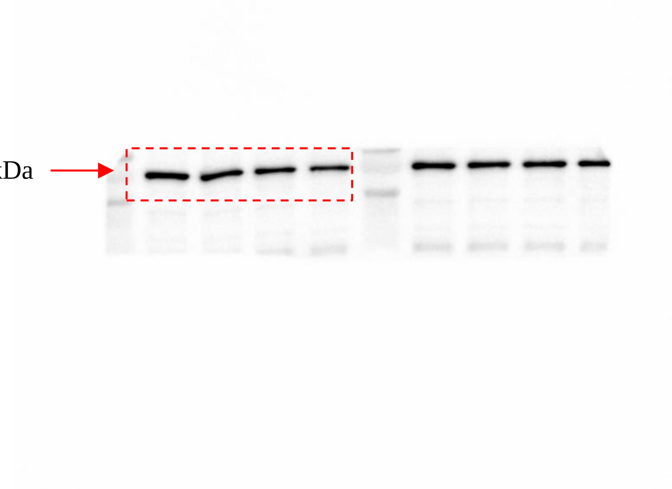

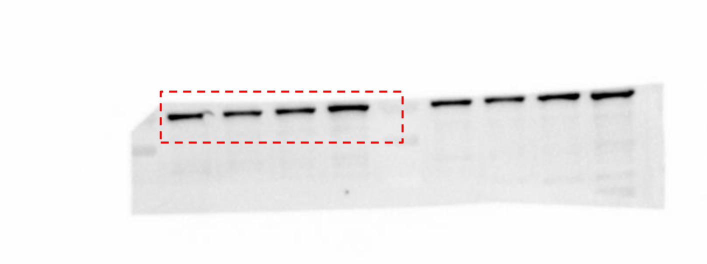

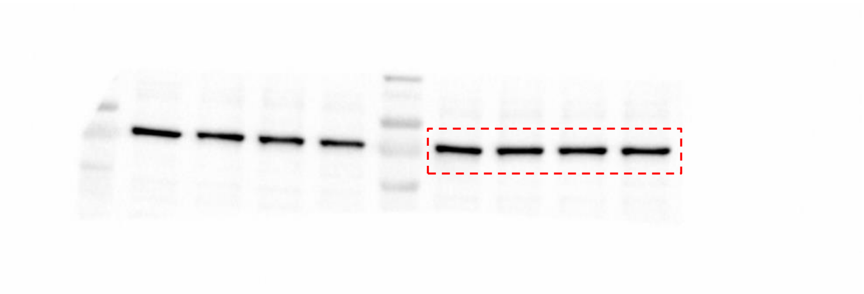

(C) Western blot original strips of AKT, p-AKT and GAPDH (Including all repeated images).

AKT 55kDa

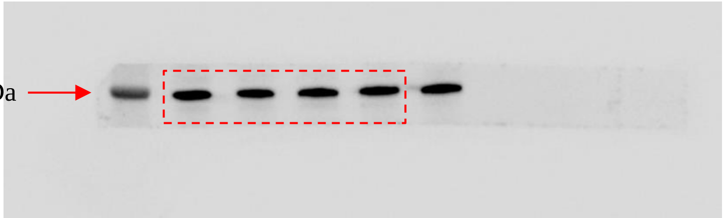

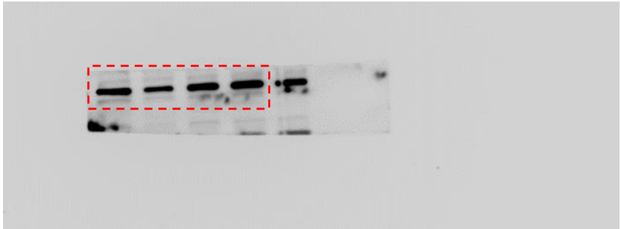

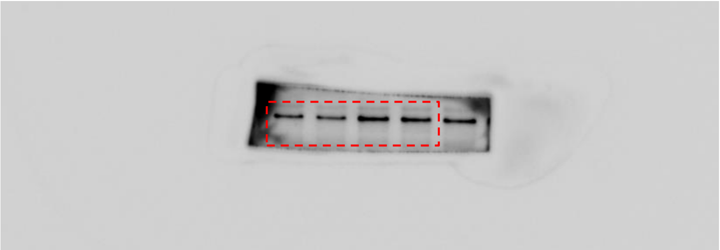

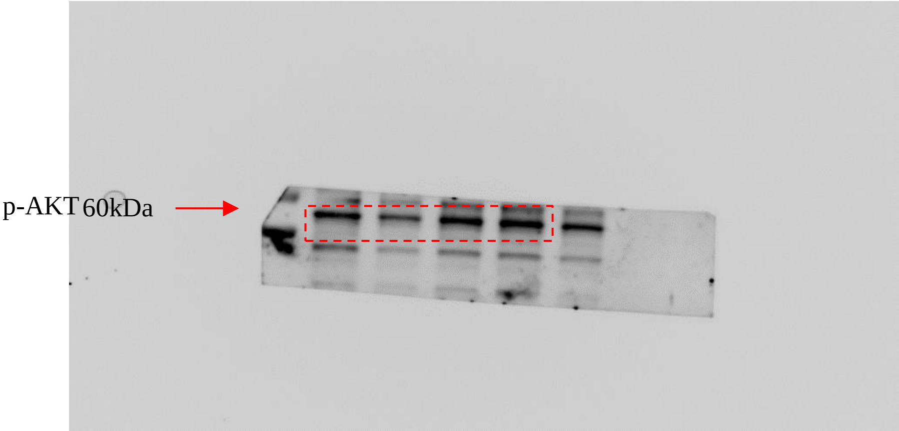
p-AKT 60kDa

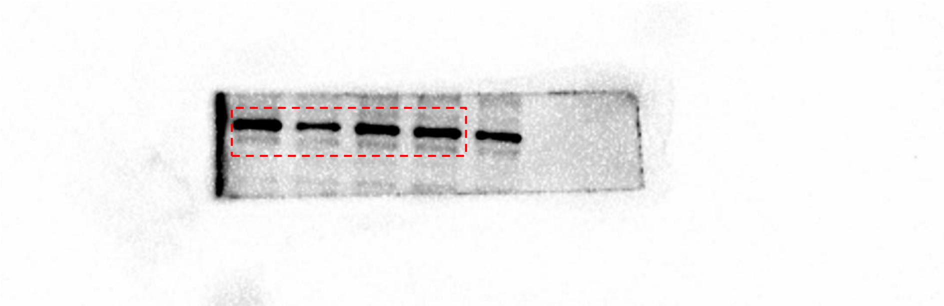

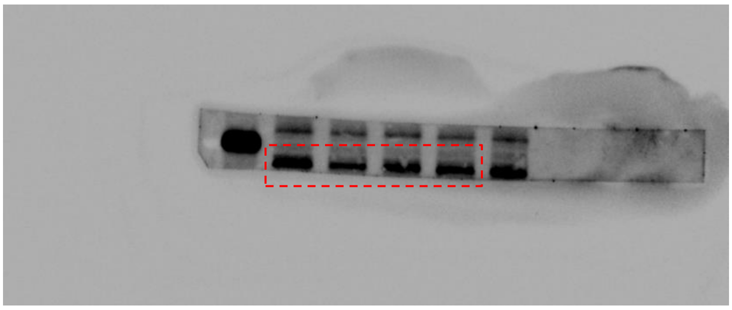

GAPDH 36kDa

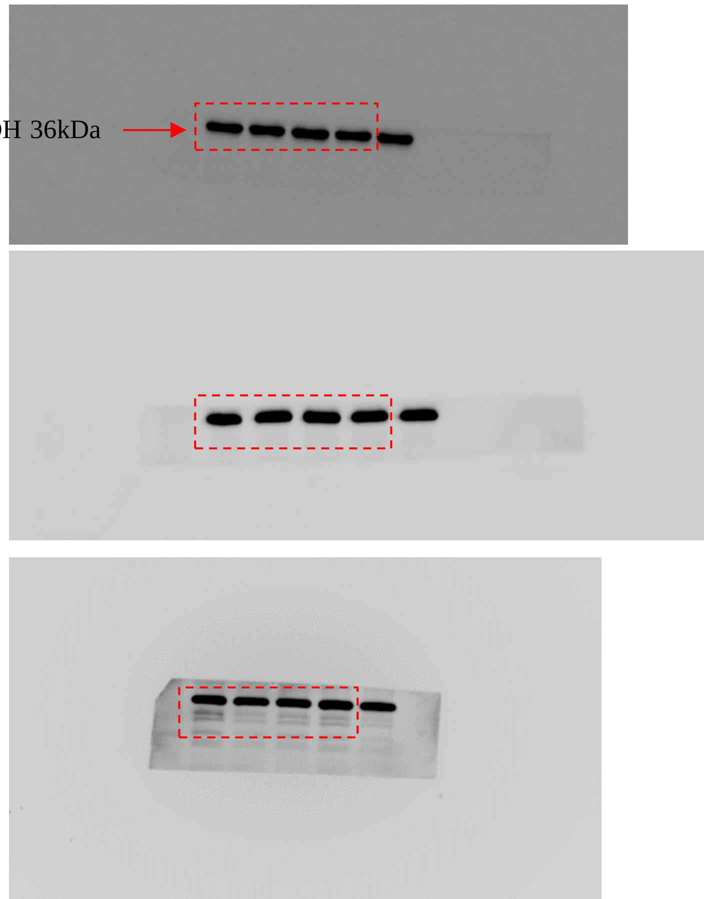

(D) Western blot original strips of PI3K, p-PI3K and GAPDH (Including all repeated images).

P-PI3K 85kDa

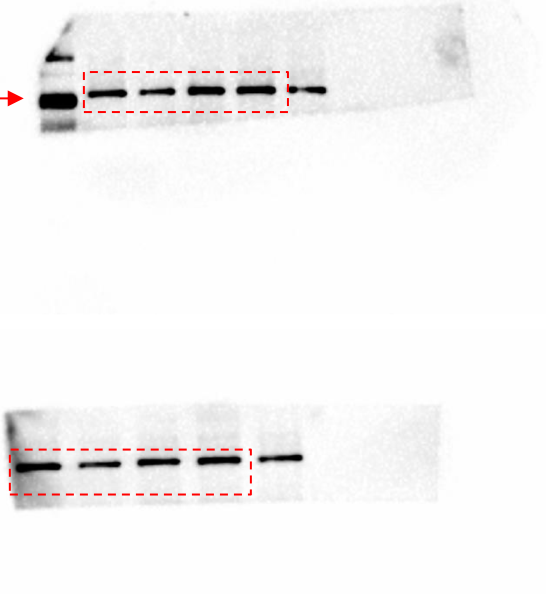

PI3K 100kDa

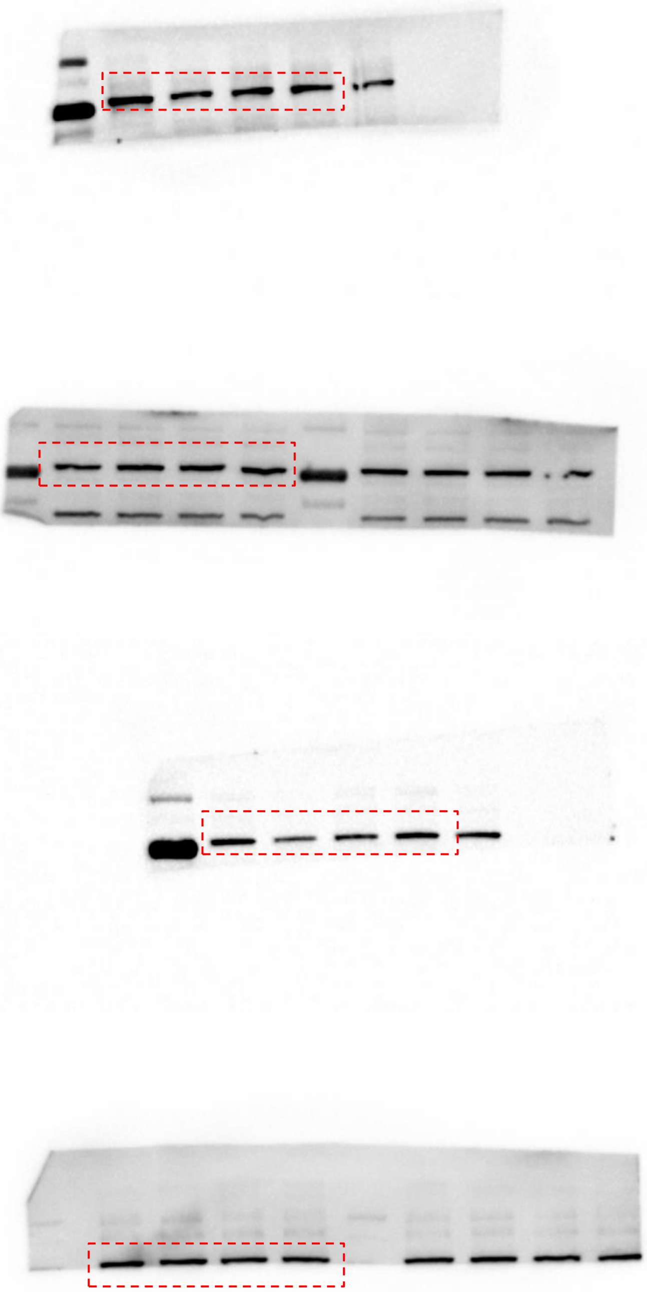

GAPDH 36kDa

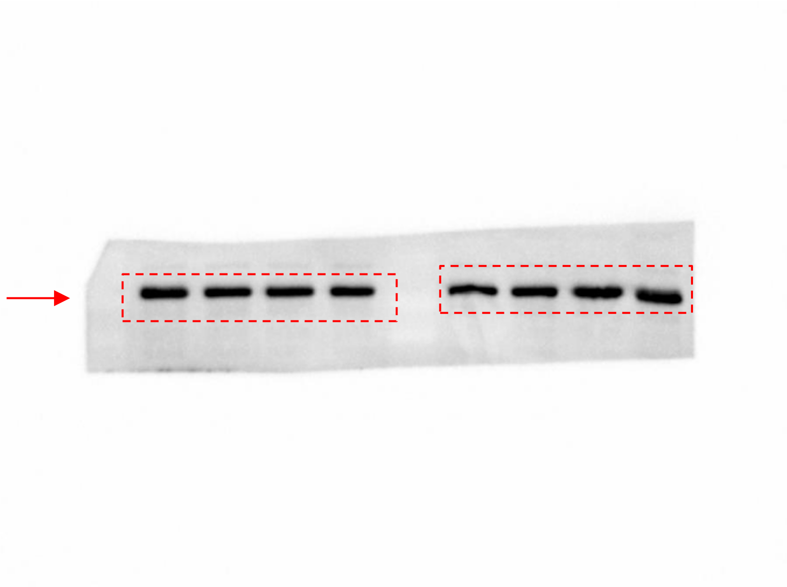

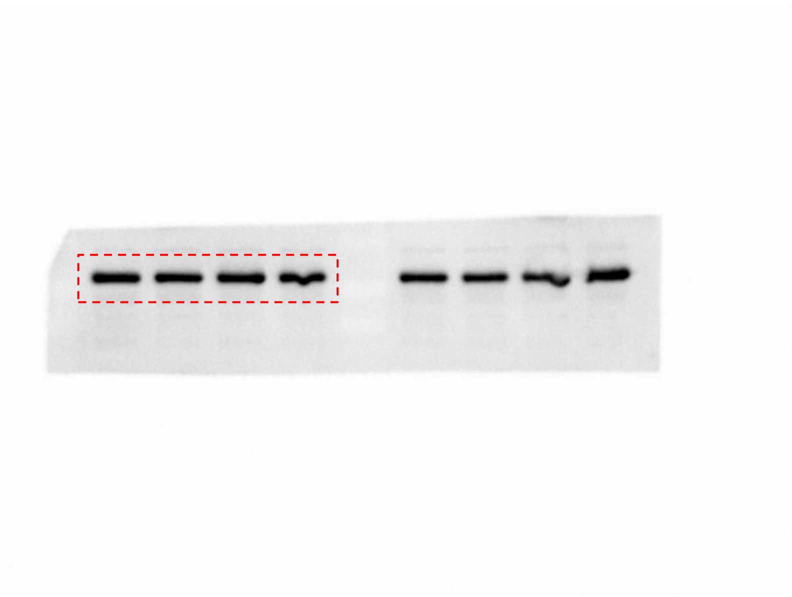

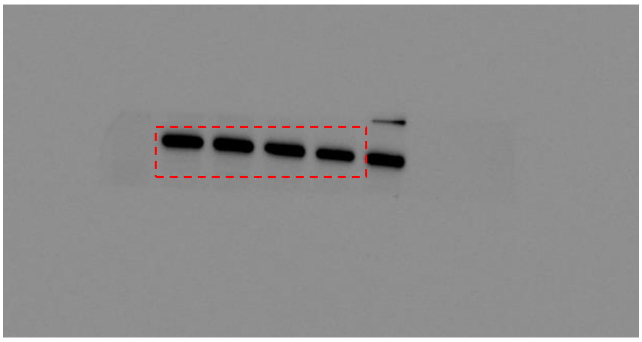

Supplement: Supplementary file 1 — Additional file 1: Supplementary Information [file 12906_2023_3925_MOESM1_ESM.pdf]
